# Nodding Syndrome: The Need to Strengthen Epilepsy Care in Onchocerciasis-Endemic Areas and to Investigate the Pathogenesis of Onchocerciasis-Associated Epilepsy

**DOI:** 10.4269/ajtmh.26-0041

**Published:** 2026-03-24

**Authors:** Amber Hadermann, Robert Colebunders

**Affiliations:** Global Health Institute, University of Antwerp, Antwerp, Belgium

In their manuscript “Nodding Syndrome: Shifting the Focus from Cause to Treatment” published in this issue of the *American Journal of Tropical Medicine and Hygiene*, Meyers et al. call for a reorientation of priorities toward improving care and treatment for persons affected by nodding syndrome (NS).^1^ We fully agree that access to comprehensive epilepsy care, which includes the provision of uninterrupted antiepileptic treatment, mental health services, nutritional support, and social protection, must be strengthened and sustained.^1^

However, it is important that such services are offered to all persons with epilepsy in onchocerciasis-endemic areas. Comprehensive studies have shown that NS is one of the clinical presentations of onchocerciasis-associated epilepsy (OAE).^2^^–^^4^ Although NS is distinguished by its characteristic head-nodding seizures, its epidemiological context is indistinguishable from that of other OAE phenotypes.^3^^–^^6^ NS represents a severe form of OAE, often associated with cognitive impairment, behavioral abnormalities, and high levels of *Onchocerca volvulus* infection.^4^^,^^7^ A case–control study in Uganda suggested that the condition predominantly occurs in children born to *O. volvulus*-infected pregnant women, as these children develop very high *O. volvulus* infection levels at a young age possibly due to parasite-specific immune tolerance transmitted from the mother during pregnancy.^8^^,^^9^

Framing NS as a rare or isolated disorder risks obscuring the much larger burden of OAE, which affects tens of thousands of individuals across multiple countries in sub-Saharan Africa.^3^^,^^10^ Epilepsy prevalences between 2-8% have been documented in onchocerciasis-endemic areas with high ongoing transmission, while the median epilepsy prevalence across sub-Saharan Africa is 1.4%.^11^^–^^23^ The highest epilepsy prevalences are observed in areas close to blackfly (*Simulium*) breeding sites.^11^^,^^20^ In those areas 70-80% of persons with epilepsy meet the criteria of OAE and several children in one household may present with epilepsy, including NS.^2^^,^^11^^,^^24^^,^^25^ Overall, it is estimated that 200,000 to 400,000 children and adolescents in sub-Saharan Africa are affected by OAE.^26^ High excess mortality due to OAE has been documented.^27^ In onchocerciasis endemic areas persons with epilepsy are seven times more likely to die than persons without epilepsy, and nearly all persons with OAE die before the age of 30 years.^11^^,^^27^

As long as the biological link between *O. volvulus* infection and epilepsy remains unknown, the association will likely continue to be questioned by policymakers, clinicians, and affected communities. This uncertainty has tangible consequences. In many endemic areas, epilepsy, including NS, continues to be attributed to witchcraft, curses, or parental neglect, thereby fuelling stigma, discrimination, and social exclusion.^28^^–^^31^ Particularly in onchocerciasis endemic areas with high ongoing or past *O. volvulus* transmission, NS cases are often clustered with other forms of epilepsy in families.^32^ Therefore, communities may consider that epilepsy is transmitted from one person to another and that such families are cursed.^29^^,^^32^ This leads to misconceptions, stigmatization, and discrimination of individuals and affected families.^29^^,^^33^ Some children with epilepsy are not allowed to sleep or eat with other children or go to school.^2^ These beliefs also lead to delays in health care-seeking and initiating antiseizure medication because affected families may first go to traditional healers.^29^^,^^31^ Clear communication that OAE, including NS, is linked to the location of the household, the area of farming and/or fishing activities, and the resulting exposure to infected blackflies will help improve acceptance of both epilepsy treatment and ivermectin distribution.^34^

Moreover, effective onchocerciasis elimination programs have been shown to substantially reduce epilepsy incidence.^12^^,^^27^^,^^35^^–^^37^ This places OAE among the few epilepsy syndromes for which primary prevention is feasible at the population level, reinforcing the need to link care for affected individuals with sustained investment in elimination programs. Any remaining uncertainty regarding causation weakens political commitment to intensified onchocerciasis elimination strategies, including optimized ivermectin distribution and complementary vector control in high-transmission settings.^34^ Etiological research is therefore not merely academic, but integral to prevention and public health action.

## The imperative of comprehensive, family-centred care.

Meyers et al. cite the example of the Hope for Human project in northern Uganda.^1^ In this project, children with NS resided in a revalidation center. This project is to be applauded for the results it obtained in improving the health and quality of life of the children included in the program. However, the program was not sustainable once external funding was no longer available because it was not integrated into the existing primary health care system.

Integration of epilepsy care into primary health care systems and providing support to families should reduce costs and ultimately be more sustainable.^38^^,^^39^ Decentralized epilepsy treatment and care, which includes task-shifting to nurses and community health workers, has been shown to be effective and should be expanded.^38^^,^^40^ Such services should include counselling to support persons with epilepsy and affected families in epilepsy self-management.

In Maridi, South Sudan, strengthening onchocerciasis elimination measures and establishing an epilepsy clinic were followed by a drastic decrease in epilepsy-related mortality rates.^41^ By preventing new-onset OAE and treating persons with epilepsy with free anti-seizure medication delivered by primary health care workers, this two-pronged approach mitigated the high mortality observed among persons with epilepsy in an onchocerciasis endemic area.^41^

## Pathogenesis, new insights.

Many causal hypotheses for NS have been proposed, but none of them, except the onchocerciasis hypothesis, meet the Bradford Hill criteria suggesting causality.^42^^–^^46^ Strong epidemiological evidence underpins the association. Based on a meta-analysis of eight population-based surveys performed in seven countries, Pion et al. estimated that, on average, the prevalence of epilepsy increased by 0.4% for each 10% increase in onchocerciasis prevalence, measured by the presence of skin microfilaria.^47^ Complementing these findings, two cohort studies in Cameroon showed a time-dependent and dose-related association between the *O. volvulus* microfilarial load in young children and the development of epilepsy later in life.^48^^,^^49^

Studies conducted prior to the introduction of mass drug administration with ivermectin (a drug that kills *O. volvulus* microfilaria (mf) but not the adult worms) detected *O. volvulus* mf in the cerebrospinal fluid (CSF) in persons without epilepsy.^50^ However, more recent studies have failed to detect *O. volvulus* nor its DNA in CSF.^51^^,^^52^ These negative results could be explained by the introduction of ivermectin, which reduces the parasitic load and may limit central nervous system (CNS) invasion. Additionally, these studies were conducted years after seizure onset, raising the possibility that *O. volvulus* mf/DNA were already cleared by the host’s immune system. However, the absence of the parasite from the CNS raises the possibility that epilepsy may be induced not by the worm as a whole, but rather by derivative components, such as excretory and secretory products, or elements of its microbiome. Disentangling these mechanisms will require a deeper understanding of the basic biology of *O. volvulus* and its intricate relationship with the host immune system. This understanding shifts the conceptional focus from hypotheses solely based on parasite presence towards mechanisms involving immune dysregulation, molecular mimicry, and/or blood-brain-barrier disruption.

A potentially important advance in understanding the complex relationship between worm and host in the development of OAE, including NS, is the discovery of diverse RNA viruses in parasitic nematodes. Of particular interest is the rhabdovirus found within *O. volvulus* and *O. ochengi*, *Onchocerca volvulus* RNA Virus 1.^53^ Notably, this virus has been shown to elicit antibody responses in vertebrate hosts, providing biological plausibility for complex host-parasite-virus interactions with systemic and potentially neurological consequences.^53^

## Toward an integrated agenda.

Rather than shifting from determination of causation to treatment, we advocate for an integrated agenda that advances both simultaneously. Continued research into etiology and pathogenesis remains essential to strengthen prevention strategies, reduce stigma, and sustain political commitment to onchocerciasis elimination. Only through such an integrated approach, combining high-quality care, effective prevention, and sustained scientific inquiry, can we hope to reduce suffering and ultimately eliminate OAE, including NS.
